# Maternal Diet Influences the Reinstatement of Cocaine-Seeking Behavior and the Expression of Melanocortin-4 Receptors in Female Offspring of Rats

**DOI:** 10.3390/nu12051462

**Published:** 2020-05-19

**Authors:** Dawid Gawliński, Kinga Gawlińska, Małgorzata Frankowska, Małgorzata Filip

**Affiliations:** Maj Institute of Pharmacology Polish Academy of Sciences, Department of Drug Addiction Pharmacology, Smętna Street 12, 31-343 Kraków, Poland; gawlin@if-pan.krakow.pl (D.G.); kingaw@if-pan.krakow.pl (K.G.); frankow@if-pan.krakow.pl (M.F.)

**Keywords:** cocaine self-administration, high-fat diet, high-sugar diet, maternal diet, pregnancy and lactation, melanocortin-4 receptor, offspring brain, rat offspring

## Abstract

Recent studies have emphasized the role of the maternal diet in the development of mental disorders in offspring. Substance use disorder is a major global health and economic burden. Therefore, the search for predisposing factors for the development of this disease can contribute to reducing the health and social damage associated with addiction. In this study, we focused on the impact of the maternal diet on changes in melanocortin-4 (MC-4) receptors as well as on behavioral changes related to cocaine addiction. Rat dams consumed a high-fat diet (HFD), high-sugar diet (HSD, rich in sucrose), or mixed diet (MD) during pregnancy and lactation. Using an intravenous cocaine self-administration model, the susceptibility of female offspring to cocaine reward and cocaine-seeking propensities was evaluated. In addition, the level of MC-4 receptors in the rat brain structures related to cocaine reward and relapse was assessed. Modified maternal diets did not affect cocaine self-administration in offspring. However, the maternal HSD enhanced cocaine-seeking behavior in female offspring. In addition, we observed that the maternal HSD and MD led to increased expression of MC-4 receptors in the nucleus accumbens, while increased MC-4 receptor levels in the dorsal striatum were observed after exposure to the maternal HSD and HFD. Taken together, it can be concluded that a maternal HSD is an important factor that triggers cocaine-seeking behavior in female offspring and the expression of MC-4 receptors.

## 1. Introduction

In the global population, every fourth person suffers from mental illnesses throughout their life, the most common of which are depression, anxiety, substance (alcohol, drugs) use disorder, and schizophrenia [1,2,3]. Substance use disorder is a chronic brain disorder associated with the uncontrolled exploration and use of drugs, leading to devastating health consequences and a destructive impact on the familial, social, and professional aspects of a patient’s life. In addition, even after long periods of abstinence, this disease is characterized by high susceptibility to relapse in response to stress and to cues or contexts associated with drugs [4].

Recent studies have focused on the contribution of a properly balanced maternal diet during intrauterine growth and early childhood in the development of the central nervous system of offspring. In fact, exposure to excessive or insufficient amounts of macronutrients (fats, sugars, proteins) can lead to morphological, molecular, and functional changes in the brains of offspring, predisposing them to the occurrence of behavioral disorders and mental diseases, such as increased impulsiveness, depression, or anxiety, which further predispose them to the risk of developing substance use disorders later in life [5,6,7,8,9]. A maternal diet rich in fat provokes increased consumption and preference for palatable but unhealthy food in offspring [10,11,12], increased nicotine and ethanol self-administration [13,14], and disturbed behavioral reactions in animals regarding the administration of psychostimulant substances (reduced locomotor activity and amphetamine-induced behavioral sensitization) [15]. In turn, a maternal diet rich in fructose or sucrose induces an increase in the amount of alcohol consumed by the offspring [16]. Together, these studies confirm the positive relationship between the maternal diet and the offspring’s susceptibility to drugs; however, there is still a lack of data on the relationship between the composition of the maternal diet and the development of cocaine use disorder (one of the most used drugs in the world among psychostimulants) [17]. Increasing knowledge about the predisposing factors for the development of mental disorders, including psychostimulant addiction, may contribute to reducing the health, social, and economic damage associated with these diseases in the future. Most literature provide data on the role of maternal obesity induced by high-calorie food on the development of the central nervous system in offspring. By limiting the modified types of diets only to pregnancy and lactation, our research will allow to indicate the period of intrauterine development and early childhood more specifically as a key factor in the development of the brain and behavioral disorders in adult offspring. Because the melanocortin system in the brain acts through melanocortin-4 (MC-4) receptors, which are located in brain regions that represent part of the reward system [18,19] and control (among other things) nutritional behavior, memory, positive enhancement, and emotions [20,21,22], these receptors were selected as a potential molecular biomarker of mechanisms underlying the cocaine susceptible phenotype in rat offspring induced by maternal nutrition. The relationship between maternal diet, MC-4 receptors and cocaine self-administration was demonstrated in recent studies by our group [23]. We showed, among other results, that a maternal high sugar-diet (HSD) significantly affects the expression of MC-4 receptors in the brains of male offspring following chronic cocaine self-administration or an abstinence period. Moreover, administration of an MC-4 receptor antagonist reduced cocaine- and cue-induced reinstatement of cocaine-seeking behavior; concurrently, male offspring that had received maternal nutrition from an HSD were more sensitive to the anti-relapsing effects of an MC-4 receptor antagonist than control male offspring [23].

In light of the above data and the small number of studies on the pathogenesis of developing addiction in females compared to studies in males, the purpose of this study was to determine the role of a maternal high-fat diet (HFD), HSD, and mixed diet (MD; rich in carbohydrate and fat) during pregnancy and lactation (critical periods in early life) on the behavioral and neurochemical changes in female offspring in the aspect of cocaine addiction. For this purpose, using the animal model of intravenous self-administration, we have comprehensively characterized the impact of a modified maternal diet on changes in the phenotype of offspring assessed at various stages of addiction: the acquisition and maintenance of cocaine addiction; abstinence; and the strength of cocaine-seeking behavior to cue- and cocaine-induced priming. In addition, at the molecular level, we evaluated the role of diet modifications during pregnancy and lactation on changes in MC-4 receptor protein expression in the synaptosomal fraction of brain structures related to cocaine addiction (the prefrontal cortex, dorsal striatum, nucleus accumbens, ventral tegmental area, and hypothalamus) in young adult female offspring.

## 2. Materials and Methods

### 2.1. Animals and Diets

All experiments were performed in accordance with the guidelines of the European Directive 2010/63/EU and were approved by the 2nd Local Institutional Animal Care and Use Committee (Maj Institute of Pharmacology Polish Academy of Sciences, Kraków, Poland; approval number 1270/2015; 42/2017). Every effort was made to minimize suffering and the number of animals used.

Wistar rats from Charles River (Germany) were housed in standard plastic rodent cages in a room maintained at 22 ± 2 °C and 55 ± 10% humidity under a 12 h light–dark cycle (lights on at 6:00 a.m.). Unless otherwise specified, animals had free access to water and food. Nulliparous female rats (200–240 g), after the acclimatization period and during the proestrus phase (smears from females were collected daily to determine the estrous cycle phase), were mated with males overnight, and pregnancy was confirmed by examining vaginal smears for the presence of sperm. Pregnant females (*n* = 10/group) were then individually housed and randomly assigned to four groups: control diet (CD; cat# VRF1; Special Diets Services, UK) or special diets purchased from Altromin (Germany): HFD (cat# C1057 mod.), HSD (cat# C1010), or MD (cat# C1011). The composition of the diets used in the study is presented in Table 1.

**Figure 1:**
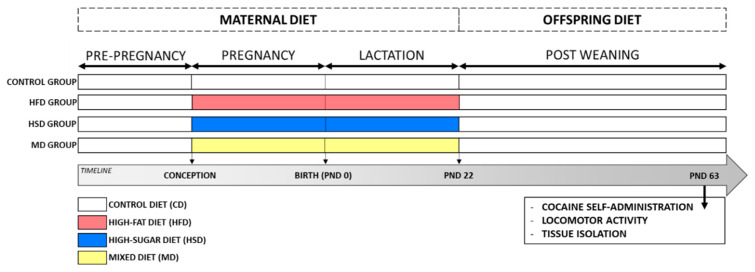
Experimental design and timeline. Dams were fed a control diet (CD) or one of the three modified diets: high-fat (HFD), high-sugar (HSD), or mixed (MD; rich in carbohydrate and fat) during pregnancy and lactation. Female offspring were divided into three cohorts and at postnatal day (PND) 63, they were assessed by tissue isolation and biochemical analyses, via the cocaine self-administration study and for locomotor activity.

Dams consumed these diets ad libitum during the gestation (21 days) and lactation periods (21 days). Dam body weight and food intake were monitored every third day. Litter sizes were normalized to 9–12 pups. After weaning, offspring were separated according to sex, housed six per cage, and fed a CD. Female offspring were used in the present study. The experimental design and timeline of the study are illustrated in Figure 1.

### 2.2. Behavioral Procedures

#### 2.2.1. Drugs

Cocaine hydrochloride (Toronto Research Chemicals, North York, ON, Canada) was dissolved in sterile 0.9% NaCl. The cocaine solution was administered intravenously or intraperitoneally. In a volume of 0.1 mL/infusion or 1 mL/kg, respectively.

#### 2.2.2. Cocaine Self-Administration Procedure

##### Catheter Implantation and Initial Lever-Press Training

At PND 53, female rats (*n* = 12 for each group) were anaesthetized with ketamine HCl (75 mg/kg; Bioketan; Biowet, Puławy, Poland) and xylazine (5 mg/kg; Sedazin; Biowet, Puławy, Poland) and chronically implanted with a silastic catheter in the external jugular vein, as described previously [24]. After surgery, meloxicam subcutaneously (0.5 mg/kg; Metacam; Boehringer Ingelheim Vetmedica GmbH, Ingelheim am Rhein, Germany) was administered, and animals were kept individually in standard home cages water and food ad libitum. Each day, catheters were flushed with 0.1 mL of saline solution containing heparin (70 U/mL; Polfa, Warszawa, Poland) or 0.1 mL of cephazolin solution (10 mg/mL; Biochemie GmbH, Kundl, Austria). After seven days of recovery, animals were food-deprived for 18–20 h (with free access to water) and were then trained to press a lever during 2-h daily sessions under the fixed ratio (FR) 1 schedule of sweetened milk reinforcement for two days. Throughout the two training days, food was limited to 70% of the rats’ free-feeding amount.

##### Cocaine Self-Administration

Female offspring at PND 63 began lever pressing for cocaine reinforcement during the 2-h daily sessions performed six days/week (maintenance) for a total of three weeks, and from that time, they were given free access to food throughout the remainder of the experiment. Rats were trained to self-administer cocaine in described previously [25] standard operant chambers (Med-Associates, Fairfax, VT, USA) in contact with an infusion pump (Model 3.33 RPM, Med-Associates, Fairfax, VT, USA) according to two experimental protocols: (a) a stable dose of cocaine (0.5 mg/kg/infusion) and an increased schedule of reinforcement (FR1–5) or (b) increased cocaine doses (0.25–1 mg/kg/infusion) and a stable FR1 schedule of reinforcement. Each schedule was completed by pressing the “active” lever resulting in a 5 s infusion of cocaine and a 5 s presentation of the stimulus complex, which consisted of an activation of the white stimulus light directly above the “active” lever and a tone from the generator (2000 Hz; 15 dB above the ambient noise level). Following each infusion, there was a 20 s time-out period during which the response was recorded but had no programmed consequences. An “inactive” lever response never activated the infusion pump.

##### Progressive Ratio Test

After the final experimental cocaine session described above, rats were tested for the self-administration of cocaine under the progressive ratio (PR) schedule of reinforcement, and this session lasted 4 h. During the PR session, the delivery of intravenous cocaine was contingent on an increasing number of responses incremented through the following progression: 1–603 [26]. Breakpoints were defined as the number of completed ratios in the series over 4 h.

##### Extinction

After cocaine self-administration (see above), the rats previously used for the two different cocaine self-administration protocols were used in the extinction training/reinstatement tests. During the extinction sessions, the animals had 1-h daily sessions with neither a cocaine delivery (exchanged to saline) nor tone and light stimuli (conditioned stimuli).

##### Reinstatement of Cocaine-Seeking

After 10 days of extinction training, animals were evaluated for the response reinstatement induced by either a conditioned cue (the tone and light associated with cocaine self-administration) or a noncontingent presentation of a self-administered reinforcement (2.5 or 10 mg/kg cocaine, intraperitoneal). The order of the cocaine priming injections was counterbalanced according to a Latin square design, and the test sessions were separated by at least 2–3 baseline sessions of extinction training. During the 2-h reinstatement tests, presses of the active lever resulted in the intravenous infusion of saline.

#### 2.2.3. Locomotor Activity

Spontaneous motor activity was recorded individually for each drug-naïve rat from the subset at PND 63 in Opto-Varimex cages (43 cm × 44 cm, Columbus Instruments, Columbus, OH, USA) and analyzed using Auto-Track software (Columbus Instruments, Columbus, OH, USA) as described previously [24]. The locomotor activity of rats was defined as horizontal activity and was presented as the distance traveled in cm during 5-, 30-, and 120-min trials.

### 2.3. Biochemical Analysis

#### 2.3.1. Brain Tissue Collection

For biochemical analysis, at PND 63, a subset of drug-naïve female offspring rats was sacrificed by rapid decapitation, and the brains were promptly removed. Relevant brain structures were dissected according to the rat brain atlas [27] and isolated on ice-cold glass plates, immediately frozen on dry ice and stored at −80 °C for enzyme-linked immunosorbent assay (ELISA) analyses. To avoid the potential effect of stress on molecular changes in the brain, animals were not fasting before decapitation. All samples were collected between 9:00–12:00 (a.m.).

#### 2.3.2. Melancortin-4 Receptor Expression

MC-4 receptor expression was determined in the synaptosomal fraction The brain tissue samples were homogenized using a sonicator (EpiShear™ Probe Sonicator; Active Motif, Carlsbad, CA, USA) in 10% (*w*/*v*) of 0.32 M sucrose HEPES buffer (containing 145 mM NaCl, 5 mM KCl, 2 mM CaCl_2_, 1 mM MgCl_2_, 5 mM glucose, and 5 mM HEPES) with a protease inhibitor cocktail (Complete, Roche, Mannheim, Germany). Later, suspended tissue was homogenized with a Dounce tissue grinder. The homogenate was centrifuged at 4 °C for 10 min at 600× *g*. The supernatant was then diluted 1:1 with 1.3 M sucrose HEPES buffer to obtain a suspension at a final concentration of 0.8 M sucrose. This suspension was further centrifuged twice in a series of washes with HEPES buffer at 4 °C for 15 min at 12,000× *g*. The supernatant was discarded each time. The pellet was suspended in RIPA buffer (containing a protease inhibitor, PMSF and 0.2% Triton X-100) and centrifuged at 4 °C for 30 min at 20,000× *g* [28]. The supernatant containing the synaptosomal fraction was frozen overnight at −20 °C and was used for further analyses the following day.

The levels of MC-4 receptors in the synaptosomal fraction of the prefrontal cortex (including the infralimbic, prelimbic, and cingulate cortices; Bregma: 5.2–2.7 mm), dorsal striatum, nucleus accumbens, ventral tegmental area, and hypothalamus were measured using ELISA kits (cat# E11964R; Wuhan EIAab Science Co., Wuhan, China) according to the manufacturer’s protocols. Duplicate aliquots of 100 μL of each sample along with MC-4 receptor standards (0, 0.312, 0.625, 1.25, 2.5, 5, 10, and 20 ng/mL) were transferred to precoated 96-well ELISA plates. The absorbance was measured at a wavelength of λ = 450 nm using a Multiskan Spectrum spectrophotometer (Thermo LabSystems, Philadelphia, PA, USA). The concentration of MC-4 receptors was calculated from a standard curve and expressed as ng/mg of protein. Bicinchoninic acid assay (BCA) protein assay kits (Thermo Scientific, Rockford, IL, USA) were used (the Pierce™ BCA Protein Assay Kit for the prefrontal cortex, dorsal striatum and nucleus accumbens or the Micro BCA™ Protein Assay kit for the ventral tegmental area and hypothalamus) to determine the protein concentrations.

### 2.4. Statistical Analysis

Animals that had problems with the catheters during the recovery or experimental periods were excluded from the data analysis. All data are expressed as the mean ± standard error of mean (SEM). Statistical analyses were performed with either one-, two-, or multi-way analysis of variance (ANOVA), with the terms of the repeated measure analysis dependent on the experiment, using Statistica version 12 software (StatSoft, Tulsa, OK, USA). Post hoc Dunnett’s or Newman–Keuls tests were used to analyze differences between group means. *p* < 0.05 was considered statistically significant.

## 3. Results

### 3.1. Maternal Body Weight, Caloric Intake and Litter Size

The effects of the modified diets on changes in dam body weight and caloric intake during pregnancy and lactation are shown in Supplemental Figure S1. A two-way ANOVA for repeated measures showed significant effects of maternal diet × day interactions (F(51, 1324) = 2.515, *p* < 0.01). We observed that dams consumed MD had lower body weight gain in the last days of lactation compared to that of the control group. In addition, it was shown that modified diet consumption resulted in a change in caloric intake during pregnancy (F(3, 36) = 4.754, *p* < 0.01) and lactation (F(3, 36) = 20.660, *p* < 0.001). Females fed an HSD consumed more calories during pregnancy (*p* < 0.05), while during lactation, significantly more calories were consumed by dams from the HFD group (*p* < 0.01), in contrast to females fed MD, in which a decrease in the average daily caloric intake was observed (*p* < 0.01; Figure S1b). At the same time, modified maternal diets did not affect the litter size (F(3, 36) = 0.873, *p* = 0.464; Figure S1c) or birth weight of female offspring (F(3, 124) = 1.585, *p* = 0.196; Figure S1d).

### 3.2. Expression of MC-4 Receptors

**Figure 2:**
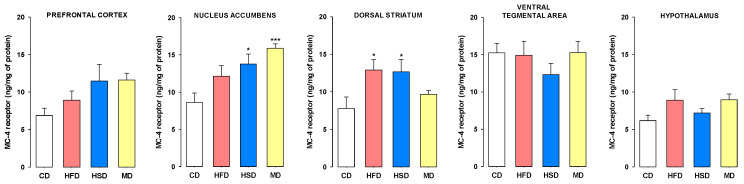
Effects of maternal diet during pregnancy and lactation on melanocortin-4 (MC-4) receptor expression in the synaptosomal fraction of the prefrontal cortex, nucleus accumbens, dorsal striatum, ventral tegmental area, and hypothalamus in female offspring rats at PND 63. The results are expressed as the mean (±SEM). *n* = 8 rats/group. Data were analyzed by one-way analysis of variance (ANOVA) followed by Dunnett’s post hoc test. * *p* < 0.05, *** *p* < 0.001 versus the control diet (CD) group. HSD, high-sugar diet; HFD, high-fat diet; MD, mixed diet.

The influence of maternal diet on the level of MC-4 receptors in the synaptosomal fraction of brain structures related to cocaine addiction (the prefrontal cortex, dorsal striatum, nucleus accumbens, ventral tegmental area, and hypothalamus) at PND 63 in the naïve female offspring was assessed (Figure 2).

We observed that modified maternal diets during pregnancy and lactation evoked changes in MC-4 receptors in the synaptosomal fraction in the nucleus accumbens (F(3, 28) = 6.604, *p* < 0.01) and dorsal striatum (F(3, 28) = 3.359, *p* < 0.05), but not in the prefrontal cortex (F(3, 28) = 2.493, *p* = 0.081), ventral tegmental area (F(3, 28) = 0.148, *p* = 0.930) or hypothalamus (F(3, 28) = 2.128, *p* = 0.119) in female offspring. Post hoc analysis showed that a maternal HSD or MD during pregnancy and lactation increased the expression of MC-4 receptors in the female nucleus accumbens (*p* < 0.05 and *p* < 0.001, respectively), and a maternal HFD or HSD increased its expression within the dorsal striatum (*p* < 0.05).

### 3.3. Locomotor Activity

Spontaneous locomotor activity, recorded for 5, 30, and 120 min, did not differ between female offspring whose mothers consumed different diets, CD, HFD, HSD, or MD, during pregnancy and lactation (Table 2).

### 3.4. Cocaine Self-Administration

The impact of maternal diets during pregnancy and lactation on cocaine self-administration in female offspring rats in two experimental protocols (stable dose of drug reinforcement and an increased reinforcement schedule, or an increased dose of the drug with a stable reinforcement schedule) was studied.

#### 3.4.1. Stable Cocaine Dose and Increased Reinforcement Schedule

**Figure 3:**
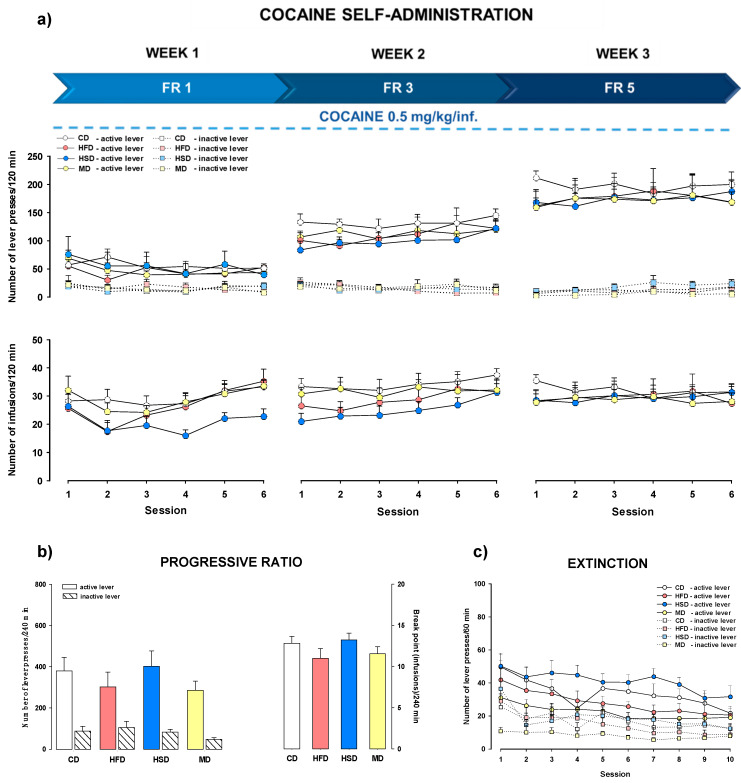
(**a**) Effects of a maternal high-fat (HFD), high-sugar (HSD), or mixed (MD) diet during pregnancy and lactation on the acquisition/maintenance of cocaine (0.5 mg/kg/infusion) self-administration with increasing fixed ratio (FR) schedules of reinforcement (FR1–5) in female offspring rats. (**b**) Effects of the modified maternal diets on cocaine self-administration with a progressive ratio schedule in female offspring day after the last session of cocaine self-administration under an increasing schedule of reinforcement (FR1–5) and a stable dose of cocaine (0.5 mg/kg/infusion). (**c**) Effects of the modified maternal diets upon self-administration extinction with a fixed dose of cocaine (0.5 mg/kg/infusion) and an increased schedule of reinforcement (FR1–5) in female offspring rats. Numbers of active and inactive lever presses and cocaine infusions are expressed as the mean (±SEM). The number of animals in each group was as follows: CD (*n* = 11), HFD (*n* = 10), HSD (*n* = 10), MD (*n* = 12). CD, control diet.

Figure 3a shows the number of active and inactive lever presses (upper panels) and the number of infusions (lower panels) during three sequential weeks with increasing FR schedules of reinforcement (FR1–5) and a stable dose of cocaine (0.5 mg/kg/infusion) for the female offspring from the CD, HFD, HSD, and MD groups. Multi-way ANOVA for repeated measures did not show significant effects of maternal diet × session × lever interactions during the eighteen days of cocaine self-administration (F(51, 1324) = 0.515, *p* = 0.998). In addition, we did not find differences between the effect of maternal diet on the number of cocaine infusions during the three weeks of self-administration, as demonstrated by a two-way ANOVA for repeated measures (F(51, 663) = 1.112, *p* = 0.280). Despite the lack of statistically significant differences, a reduced amount of infusions can be seen in the first and second weeks of self-administration in female offspring exposed to a maternal HSD.

#### 3.4.2. Increased Cocaine Dose and Stable Reinforcement Schedule

**Figure 4:**
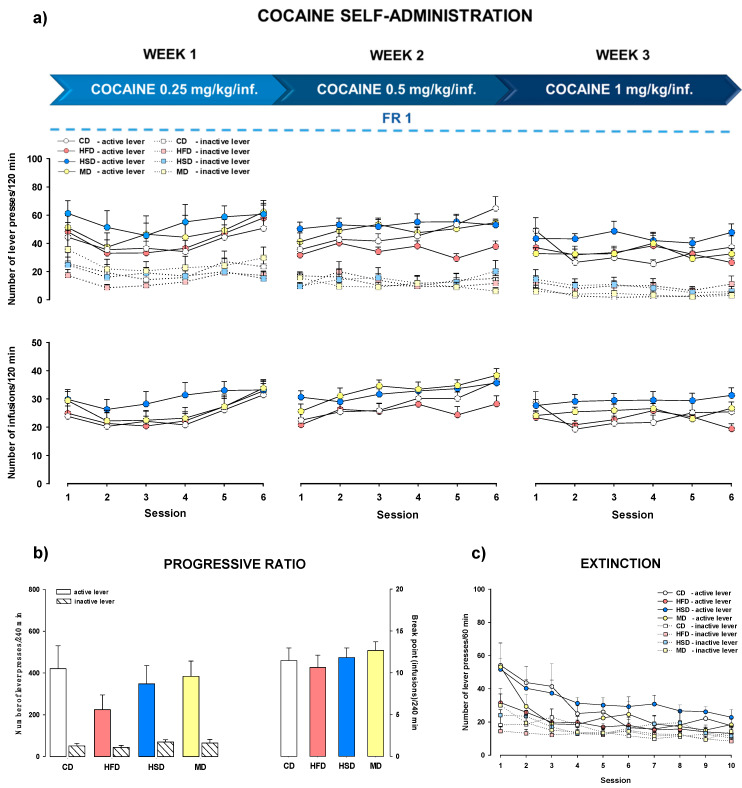
(**a**) Effects of a maternal high-fat (HFD), high-sugar (HSD), or mixed (MD) diet during pregnancy and lactation on the acquisition/maintenance of cocaine (0.25–1 mg/kg/infusion) self-administration with a stable FR1 schedule of reinforcement in female offspring rats. (**b**) Effects of the modified maternal diets on cocaine self-administration with a progressive ratio schedule in female offspring day after the last session of cocaine self-administration with a stable FR1 schedule of reinforcement and an increasing dose of cocaine (0.25–1 mg/kg/infusion). (**c**) Effects of the modified maternal diets on self-administration extinction with increased doses of cocaine (0.25–1 mg/kg/infusion) and a stable FR1 schedule of reinforcement in female offspring rats. Numbers of active and inactive lever presses and cocaine infusions are expressed as the mean (±SEM). The number of animals in each group was as follows: cocaine self-administration CD (*n* = 12), HFD (*n* = 11), HSD (*n* = 11), MD (*n* = 12); extinction training CD (*n* = 11), HFD (*n* = 10), HSD (*n* = 10), MD (*n* = 12). CD, control diet.

The number of active and inactive lever presses (upper panels) and the number of cocaine infusions (lower panels) for the increasing doses of cocaine (0.25, 0.5 and 1 mg/kg/infusion) with a stable FR1 reinforcement schedule during the entire experiment are shown in Figure 4a. We observed that the active and inactive lever presses of female offspring whose mothers were fed modified diets during pregnancy and lactation did not differ significantly from the lever presses conducted by the control diet group in this protocol. Multi-way ANOVA for repeated measures did not reveal significant maternal diet × session × lever interactions during the eighteen sessions of cocaine self-administration (F(51, 1428) = 0.896, *p* = 0.682). A two-way ANOVA for repeated measures also showed no significant changes in the number of cocaine infusion (F(51, 714) = 0.989, *p* = 0.497) in offspring.

### 3.5. PR Schedule of Cocaine Reinforcement

The female offspring from the HFD, HSD, and MD groups did not differ from the control group in the break point during cocaine self-administration under the PR reinforcement schedule following an increasing schedule of reinforcement and a stable dose of cocaine (F(3, 39) = 1.222, *p* = 0.315). Animals did not differ in active and inactive lever presses during cocaine (0.5 mg/kg/infusion) self-administration in the PR protocol (F(3, 78) = 0.637, *p* = 0.594; Figure 3b).

Maternal exposure to HFD, HSD, or MD did not change behavioral readouts in female offspring in cocaine (1 mg/kg/infusion) self-administration in the PR scheme following a stable FR1 reinforcement schedule and an increasing dose of cocaine (number of cocaine infusions (F(3, 42) = 0.406, *p* = 0.749); number of active and inactive lever presses (F(3, 84) = 0.804, *p* = 0.495; Figure 4b).

### 3.6. Extinction Training

Extinction training was introduced to all rats after cocaine self-administration. After substituting saline for cocaine, a progressive drop in lever responses was observed over the extinction sessions for the CD, HFD, HSD, and MD groups. In the female offspring trained to self-administer cocaine (0.5 mg/kg/infusion) with the increased FR1–5 schedule of reinforcement, a multi-way ANOVA for repeated measures did not show significant effects of maternal diets on extinguished active lever pressing (F(27, 702) = 0.545, *p* = 0.972; Figure 3c). Rats from mothers fed different diets did not differ in their extinction of active lever responses (F(27, 702) = 0.442, *p* = 0.994) following cocaine (from 0.25 to 1 mg/kg/infusion) with the FR1 reinforcement schedule (Figure 4c).

### 3.7. Reinstatement of Cocaine-Seeking Behavior

Following 10 days of extinction training, all groups of female rats were tested for response reinstatement induced by the cocaine-associated cue or cocaine (2.5 or 10 mg/kg, intraperitoneal).

#### 3.7.1. Relapse after Stable Cocaine Dose and Increased Reinforcement Schedule

**Figure 5:**
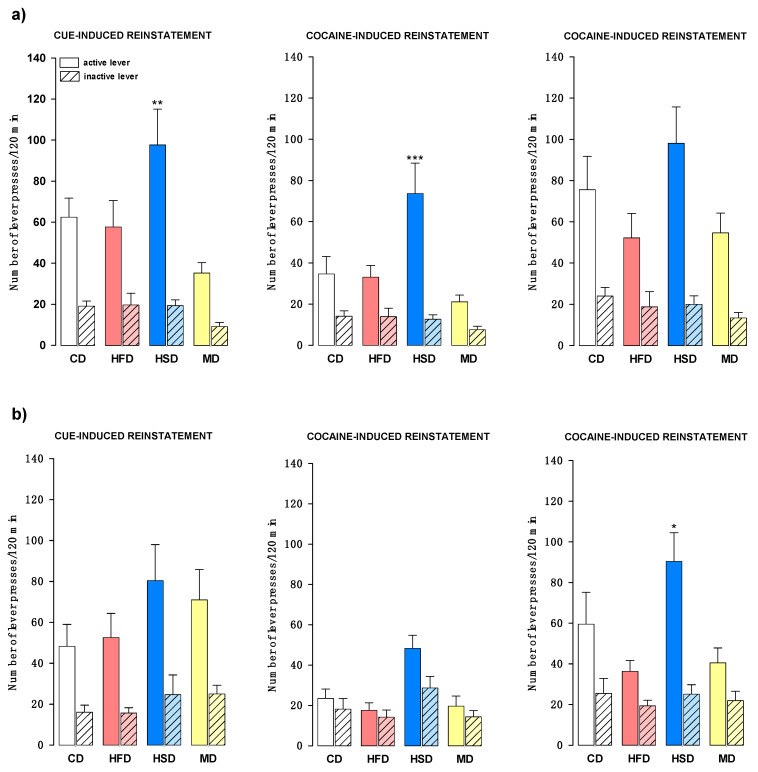
Effects of a maternal high-fat (HFD), high-sugar (HSD), or mixed (MD) diet during pregnancy and lactation on reinstatement of the cocaine-seeking behavior induced by the cue (CUE; tone + light) and the drug in female rat offspring. Drug-induced reinstatement was triggered by the administration of cocaine (2.5 or 10 mg/kg, i.p.). (**a**) Tests were performed after cocaine (0.5 mg/kg/infusion) self-administration with an increasing schedule of reinforcement (FR1–5) and 10 days of extinction training. (**b**) Tests were performed after cocaine (0.25–1 mg/kg/infusion) self-administration with a stable FR1 schedule of reinforcement and 10 days of extinction training. The numbers of active and inactive lever presses are expressed as the mean (±SEM). The number of animals each group was as follows: CD (*n* = 11), HFD (*n* = 10), HSD (*n* = 10), MD (*n* = 12). Data were analyzed by two-way ANOVA and the post hoc Newman–Keuls test. * *p* < 0.05, ** *p* < 0.01, *** *p* < 0.001 versus the control diet (CD) group.

Figure 5a shows the number of active and inactive lever presses in offspring that were previously subjected to self-administered cocaine (0.5 mg/kg/infusion) with the increased FR schedule of reinforcement. Statistical analysis demonstrated that in female offspring, modified maternal diets changed cue-induced reinstatement (F(3, 78) = 3.453, *p* < 0.05) as well as cocaine-induced reinstatement for a cocaine dose of 2.5 mg/kg (F(3, 78) = 4.670, *p* < 0.01). Furthermore, there was no significant difference in the strength of the recurrence of cocaine-seeking behavior between the CD group and the tested diets for cocaine doses of 10 mg/kg (F(3, 78) = 1.661, *p* = 0.182). Post hoc tests indicated an increase in active lever responses only in female offspring from the HSD group upon re-exposure on cue or intraperitoneal cocaine administration at a dose of 2.5 mg/kg (*p* < 0.01 and *p* < 0.001, respectively).

#### 3.7.2. Relapse after Increased Cocaine Dose and Stable Reinforcement Schedule

The number of active and inactive lever responses in animals that were previously trained to self-administer increasing cocaine doses (0.25–1 mg/kg/infusion) with a stable FR1 schedule of reinforcement is shown in Figure 5b. We found that a maternal HFD, HSD, and MD did not significantly change the female offspring relapse strength after re-exposure to cocaine-associated cues (F(3, 78) = 1.895, *p* = 0.448) and cocaine (2.5 mg/kg) compared to that of the control animals (F(3, 78) = 1.990, *p* = 0.122). However, differences in active lever presses were observed after cocaine was administered at a dose of 10 mg/kg (F(3, 78) = 3.043, *p* < 0.05). The HSD female offspring pressed the active lever more times during the test (*p* < 0.05) compared to the number of presses performed by the CD group.

## 4. Discussion

In this study, we showed that a modified maternal diet during pregnancy and the lactation period is an important factor inducing impairments at the behavioral and neurochemical levels and a cocaine-seeking prone phenotype in female offspring. We focused on females due to the observed differences in addiction to psychoactive substances in relation to sex (e.g., women are more likely to relapse than men) [29] as well as the smaller number of studies assessing the effect of maternal nutrition on behavioral changes in female offspring compared to the number of studies regarding male offspring. The results of this work are the first to indicate that a modification in the composition of maternal diet, particularly an increased amount of carbohydrates (mainly sucrose) consumed during fetal development and early childhood, results in an increased relapse of cocaine-seeking behavior and in increased levels of MC-4 receptors in selected brain structures in female offspring.

Human research indicates that both obesity and the consumption of an HFD increases total food consumption and the risk of alcohol [30,31] and nicotine addictions [32]. Recently, an increasing number of preclinical studies focused mainly on maternal HFDs have proven that nutrition may predispose offspring to an increased intake of palatable food [10,11,33], drinking alcohol [16,34,35], nicotine [14], or simultaneous ethanol and nicotine [13] self-administration. In this paper, a series of behavioral tests using an animal model of intravenous cocaine self-administration to determine the female offspring phenotype after exposure to modified maternal diets were performed. We showed that the predisposition to cocaine self-administration in female rats from a CD, HFD, HSD, or MD groups was similar and did not depend on the schedule of reinforcement or the dose range of cocaine. In fact, the animals did not differ significantly in the number of presses on the active and inactive levers or the number of drug infusions during the acquisition/maintenance phase in either the cocaine motivational scoring protocol (FR1–5) while testing the rewarding effects of the drug (cocaine 0.25–1 mg/kg/infusion) or during the following test using the PR schedule to assess animals’ motivation. Moreover, following three weeks of exposure to cocaine self-administration, no changes in female behavior were noted during the drug abstinence period. In other words, modified maternal diets during pregnancy and lactation did not influence either the rewarding or motivational aspects of cocaine intake in female offspring.

Substance use disorder is a debilitating chronic brain disease characterized by high susceptibility to relapse in response to stress or stimuli associated with previous drug use, even after a long period of abstinence [4]. Approximately 40–60% of human addicts entering therapy return to using addictive substances within the first year of treatment [36]. Due to the serious problem of relapse concerning effective addiction therapy, our main finding was that in comparison to the control groups, the females consuming an HSD, depending on the protocol of cocaine self-administration, evoked a higher reinstatement following re-exposure to the cue associated with the previous cocaine infusions or the presentation of the cocaine-priming dose (10 mg/kg). Interestingly, cocaine at a dose of 2.5 mg/kg, which normally does not cause relapse (subthreshold dose), also significantly enhanced the active lever presses in the female offspring of mothers that had consumed an HSD. It seems, therefore, that the increased potency of cocaine relapse in offspring exposed to a maternal HSD may depend not only on the conditions of individual development, but also on the pattern of drug use before abstinence. Moreover, in both schedule protocols, there was no change in the rats’ responses to the inactive lever, which suggests the specificity of the observed behavioral responses. Additionally, naïve female offspring from diet-exposed groups showed similar spontaneous locomotor activity, which means that the rats’ motor activity was not affected by the maternal diets and that the observed changes in cocaine-seeking behavior are specific. Our findings showed that the effect of a maternal HSD during pregnancy and lactation on the severity of a cocaine relapse is not dependent on sex, as a similar increase in the reinstatement of drug-seeking behavior was found in male offspring [23].

Behavioral studies emphasize that prenatal exposure to cocaine causes increased self-administration of cocaine [37,38,39] or alcohol [40] in offspring. Data from human observations indicate that exposure to cocaine in utero leads to the earlier and more frequent use of drugs (cocaine, marijuana, alcohol, and nicotine) by offspring in adolescence and early adulthood [41,42,43,44]. On the other hand, the literature provides evidence that the consumption of a natural reward, such as sugar, manifests similar neurochemical effects in the brain as the use of most addictive psychoactive substances. Within the central reward system, an increased dopamine level was observed [45,46,47,48], as was the altered expression of dopamine receptors [49,50,51] or the adaptive reduction of dopamine levels as a result of chronic exposure to a sweet, natural reward [52]. In addition, behavioral changes characteristic of drug addiction were observed similar to the neurochemical responses to natural rewards and psychoactive substances. These included, among others, increased sugar consumption [53] or increased animal responses to a conditional stimulus associated with prior sucrose self-harvesting [54] after a period of abstinence. Hence, the abuse of tasty foods or drugs is not the only way to induce similar neurochemical and behavioral changes. Preclinical studies emphasize that exposure to a modified maternal diet during intrauterine development, as well as addictive substances, also significantly affects the offspring reward system being formed and its behavior later in life. Exposure to a diet rich in energy (e.g., an HFD or western-type diet) during pregnancy and lactation induced increased cocaine-induced place preference in the conditioned place preference (CPP) test, alcohol consumption, sensitivity to amphetamine administration, and preference for fat, and at the molecular level changes in dopaminergic brain signaling in juvenile and adult offspring [10,12,35,55]. There is also evidence of the interaction of natural rewards (e.g., sucrose) and addictive substances such as cocaine [56,57,58], methamphetamine [59], or alcohol [60]. For example, sensitization of behavioral responses occurs following cocaine administration and prolonged activity involving addictive substances in rats having intermittent access to granulated sucrose compared to the behavioral responses in animals consuming only standard laboratory feed [56]. This may suggest that maternal exposure to an increased amount of sucrose relative to a CD during pregnancy and early childhood, which is crucial for normal brain development, may lead to hypersensitivity between the natural reward (sugars) and the psychostimulants in offspring. Hence, female offspring of mothers that consumed an HSD may be more sensitive to conditional and unconditional stimuli associated with previous positive enhancements experienced during cocaine self-administration.

One of the neurochemical mechanisms that can potentially explain the offspring’s behavior to cocaine may be associated with changes in the melanocortin system induced by a modified maternal diet. In fact, female offspring of mothers that consumed an HSD demonstrated increased cocaine-seeking behavior irrespective of the protocol, and the drug-naïve young adults female originating from mothers that consumed an HSD showed an upregulation of MC-4 receptors in the synaptosomal fraction of the nucleus accumbens and dorsal striatum, the brain regions linked to drug-seeking behavior [61,62]. Similarly, increased levels of accumbal MC-4 receptors were observed in female offspring exposed to an MD or in the dorsal striatum in the HFD group, but such changes did not provoke behavioral changes. This indicates the complexity of the factors involved in cocaine-seeking behavior or that behavioral changes manifest only when disturbance within MC-4 receptors occurs in several structures of the reward system. The simultaneous change in the expression of MC-4 receptors within the nucleus accumbens and dorsal striatum may lead to different activities of these structures than that observed in members of the CD group after exposure to conditional and unconditional stimuli, which may contribute to the observed intensification of the reinstatement of cocaine-seeking behavior in these animals. Previous studies assessing the effect of HFD intake by mothers before conception and during pregnancy and lactation showed reduced MC-4 mRNA expression in the female hypothalamus of offspring immediately after lactation (PND 20) [63]. Our findings demonstrate that switching to a CD after weaning, on one hand, can restore the basal level of MC-4 receptors in the hypothalamus, but the maternal diet leading to distant neurochemical consequences occurring in the nucleus accumbens and dorsal striatum does not disappear during the lifetime of the offspring.

The changes within the MC-4 receptors observed in the rat brain regions associated with the dopamine mesocorticolimbic system seem to be particularly important, as the brain structure is strongly associated with psychostimulant reward effects [64] and is involved in cue-, cocaine-, or stress-inducing reinstatement [65,66], supplementing existing knowledge about the interaction of psychoactive substances and MC-4 receptors. Recent studies have shown a decrease in the activity of agouti-related protein (AgRP) and proopiomelanocortin (POMC) neurons in the hypothalamus after the administration of cocaine, amphetamine, and nicotine, which suggests that not only reward pathways but also neuronal pathways associated with maintaining homeostasis affect the enhancement of these substances [67]. Typically, passively administered cocaine resulted in an increase in MC-4 receptor mRNA levels in the striatum, hippocampus, and hypothalamus [68,69]. Moreover, administration of the MC-4 receptor antagonist SHU-9119 to the nucleus accumbens reduced low-dose cocaine self-administration (0.125–0.25 mg/kg/infusion), cocaine-induced CPP, and the reinstatement of cocaine-seeking behavior [69]. On the other hand, the blockade of central MC-3/4 receptors with AgRP resulted in the blockade of acute and sensitized locomotor responses to cocaine [70]. Our recent data showed differences in the levels of MC-4 receptors within the prefrontal cortex, nucleus accumbens, dorsal striatum, and amygdala between CD and HSD male offspring after three weeks of cocaine self-administration and 10 days of extinction [23]. The data suggest that a maternal HSD during pregnancy and lactation interferes with the adaptive mechanisms in the brains of male offspring, during which abstinence can restore melanocortin signaling homeostasis after cocaine exposure. Current evidence suggests that MC-4 receptor activity may affect behavioral aspects of the cocaine response through interaction and modulation of the dopaminergic system [71,72,73,74,75].

In summary, the described results emphasize the important role of a maternal diet rich in sugars during fetal development and early childhood in the predisposition of female offspring to cocaine-seeking behavior in adult life. Moreover, an altered amount of macronutrients in the maternal diet disrupts the proper expression of MC-4 receptors in brain structures involved in cocaine relapse in female offspring, thus leading to a stronger response to exposure to conditioned and unconditioned stimuli combined with earlier cocaine self-administration.

## Figures and Tables

**Table 1:** Macronutrient profiles (expressed as a percentage of energy) and energy values of the control and modified diets used in this study.

	Control Diet (CD)	High-Fat Diet (HFD)	High-Sugar Diet (HSD)	Mixed Diet (MD)
**Carbohydrate** **Sucrose**	65% 4.6%	25%	70% 44%	56% 18%
**Fat**	13%	60%	12%	28%
**Protein**	22%	15%	18%	16%
**Total energy**	3.4 kcal/g	5.3 kcal/g	3.8 kcal/g	3.9 kcal/g

**Table 2:** Locomotor activity of female offspring at postnatal day 63.

		CD	HFD	HSD	MD	
**Distance traveled (cm)**	5 min	1064.93 ± 63.75	1031.16 ± 56.43	1112.13 ± 71.78	1018.53 ± 60.86	F(3, 44) = 0.435, *p* = 0.729
	30 min	3130.32 ± 214.53	3332.69 ± 223.62	3210.91 ± 145.59	3070.44 ± 217.69	F(3, 44) = 0.313, *p* = 0.816
	120 min	5262.90 ± 380.66	4979.64 ± 284.89	5615.37 ± 603.51	5392.65 ± 562.38	F(3, 44) = 0.311, *p* = 0.817

Distance traveled (cm) was measured after 5, 30, and 120 min in female offspring whose mothers were fed a high-fat (HFD), high-sugar (HSD), or mixed diet (MD) during pregnancy and lactation. The results are expressed as the mean (±SEM). *n* = 12 rats/group. Data were analyzed by one-way analysis of variance (ANOVA) versus the control diet (CD) group.

## Supplementary Materials

The following are available online at https://www.mdpi.com/2072-6643/12/5/1462/s1, Figure S1: Effects of a high-fat (HFD), high-sugar (HSD), or mixed (MD) diet on (a) maternal body weight changes (as a percentage of weight gain compared to start weight) and (b) average daily energy intake during pregnancy and lactation. Effects of the modified maternal diets on (c) litter size and (d) birth body weight of female offspring.
