# Supplementary material for: Maternal Diet Influences the Reinstatement of Cocaine-Seeking Behavior and the Expression of Melanocortin-4 Receptors in Female Offspring of Rats
Source: Nutrients. 2020 May 19;12(5):1462. doi: 10.3390/nu12051462 (PMC7284813; doi:10.3390/nu12051462)
Supplement: Supplementary file 1 [file nutrients-12-01462-s001.pdf]

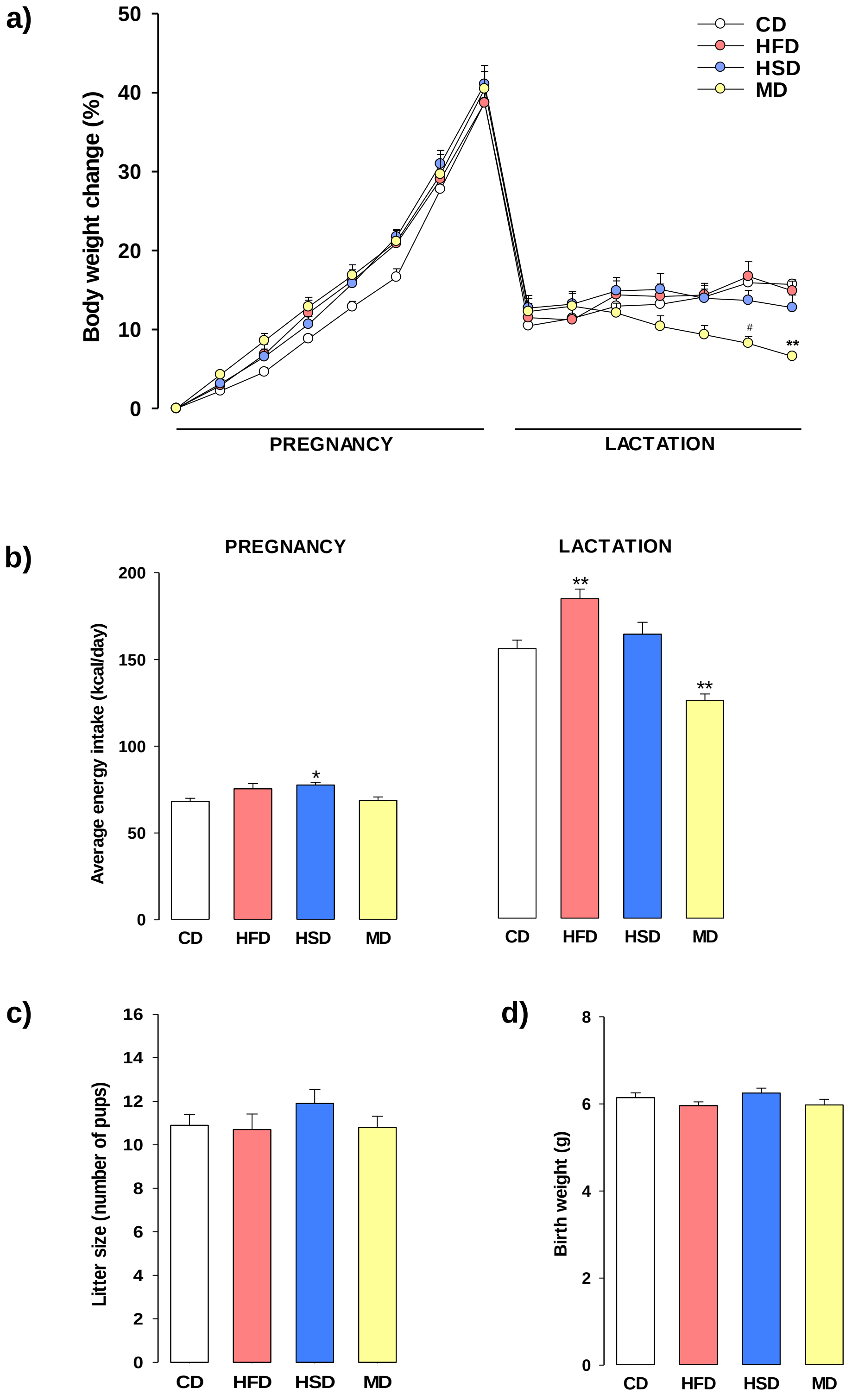

Figure S1. Effects of a high-fat (HFD), high-sugar (HSD), or mixed (MD) diet on **(a)** maternal body weight changes (as a percentage of weight gain compared to start weight) and **(b)** average daily energy intake during pregnancy and lactation. Effects of the modified maternal diets on **(c)** litter size and **(d)** birth body weight of female offspring. The results are expressed as the mean (±SEM). N = 10 rats/group for dams and n = 32 rats/group for female offspring. Data were analyzed by one- or two-way ANOVA followed by Dunnett's or Newman-Keuls post hoc tests. $^{\#}p = 0.06$, $^{*}p < 0.05$, $^{**}p < 0.01$ versus the control diet (CD) group.
